# MatrixQCvis: shiny-based interactive data quality exploration for omics data

**DOI:** 10.1093/bioinformatics/btab748

**Published:** 2021-11-12

**Authors:** Thomas Naake, Wolfgang Huber

**Affiliations:** Genome Biology Unit, European Molecular Biology Laboratory, Heidelberg 69117, Germany; Genome Biology Unit, European Molecular Biology Laboratory, Heidelberg 69117, Germany

## Abstract

**Motivation:**

First-line data quality assessment and exploratory data analysis are integral parts of any data analysis workflow. In high-throughput quantitative omics experiments (e.g. transcriptomics, proteomics and metabolomics), after initial processing, the data are typically presented as a matrix of numbers (feature IDs × samples). Efficient and standardized data quality metrics calculation and visualization are key to track the within-experiment quality of these rectangular data types and to guarantee for high-quality datasets and subsequent biological question-driven inference.

**Results:**

We present MatrixQCvis, which provides interactive visualization of data quality metrics at the per-sample and per-feature level using R’s shiny framework. It provides efficient and standardized ways to analyze data quality of quantitative omics data types that come in a matrix-like format (features IDs × samples). MatrixQCvis builds upon the Bioconductor SummarizedExperiment S4 class and thus facilitates the integration into existing workflows.

**Availability and implementation:**

MatrixQCVis is implemented in R. It is available via Bioconductor and released under the GPL v3.0 license.

**Supplementary information:**

Supplementary data are available at *Bioinformatics* online.

## 1 Introduction

**Fig. 1.**
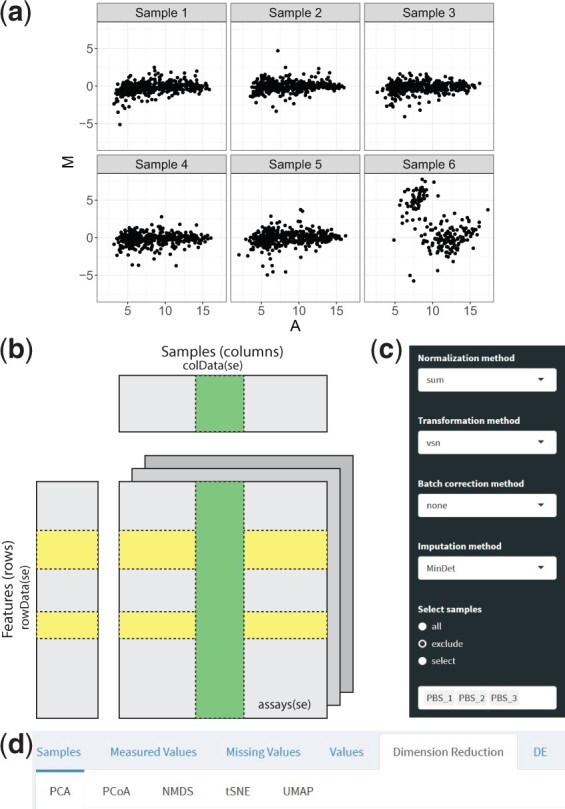
Examples of MatrixQCvis functionality and user interface. (**a**) MA plot of human plasma proteomics samples identifying a dependence between *M* and *A* values for Sample 6 indicating problems with its data. More visualizations using the clinical datasets of Jiang *et al.* (2019) and Brueffer *et al.* (2018) are shown in the Supplementary Material. (**b**) MatrixQCvis builds upon the SummarizedExperiment S4 class, a container for assay data (e.g. proteomics intensity values) and associated metadata on the features and samples. The figure is adjusted from the vignette of the SummarizedExperiment package. (**c**) Sidebar panel. MatrixQCvis enables to interactively normalize, transform, perform batch correction and impute the dataset. Furthermore, samples can be excluded or selected. In the shown example, phosphate-buffered saline samples are excluded. (**d**) Main panel. Navigation within MatrixQCvis is realized by browsing through tabs. Each visualization is embedded within a dedicated tab

Initial first-line data quality assessment is an integral part of data analysis for subsequent biological question-driven inference. To ensure facile exploration of data quality, we developed MatrixQCvis, implemented in the R programming language. MatrixQCvis provides shiny-based interactive visualization and quantification of data quality metrics at the per-sample and per-feature level. It is broadly applicable to quantitative omics data types that come in a matrix-like format (features × samples). It enables the detection of low-quality samples, outliers, drifts and batch effects in datasets. Visualizations include boxplots and violin plots of the (count or intensity) values, mean versus standard deviation plots, MA plots (see Fig.  1a) and Hoeffding’s D statistic (non-parametric measure of independence between M and A), empirical cumulative distribution function plots, visualizations of the distances between samples and multiple types of dimension reduction plots. Furthermore, MatrixQCvis facilitates differential expression analysis based on the limma (moderated *t*-tests, Ritchie *et al.*, 2015) and proDA (Wald tests, Ahlmann-Eltze and Anders, 2019) packages.

Similarly to the iSEE package (Rue-Albrecht *et al.*, 2018), MatrixQCvis builds upon the widely used Bioconductor SummarizedExperiment S4 class (see Fig.  1b) and thus facilitates the integration into existing workflows. Compared to iSEE, which provides a general interface for exploring data in a SummarizedExperiment object, MatrixQCvis focuses on the upstream, initial first-line, data quality control steps and incorporates dedicated visualization capabilities for assessing data quality, although overlaps exist (e.g. dimension reduction plots). Several software packages exist that center around the assessment of data quality: among others, arrayQualityMetrics (Kauffmann *et al.*, 2009), initially developed more than 10 years ago for the quality assessment of microarrays, that creates automatic reports of the data quality. Contrary to arrayQualityMetrics, which is built upon outdated visualization libraries, MatrixQCvis uses the shiny (Chang *et al.*, 2021) framework and provides a high number of interactive visualizations to explore the data. MeTaQuaC (Kuhring *et al.*, 2020), a recently published R package, is dedicated to metabolomics data analysis, accepts either the Biocrates data output or a generic file format and creates a static quality control report. On the other hand, MatrixQCvis is not restricted to a specific technology and centers around interactive exploration of data quality.

We highlight the usability and functionality of the MatrixQCvis package in applications of clinical proteomics and transcriptomics studies in the Supplementary Material, using the datasets of Jiang *et al.* (2019) and Brueffer *et al.* (2018).

## 2 Usage scenario and user interface

Many tools and software packages exist that directly process raw data and translate these to biological discovery, but offer limited capabilities for data quality control. However, data quality control is a necessary step in omics data processing to identify samples with poor intensities and low signal-to-noise ratio, biases and outliers, batch and confounding effects or drifts in calibration. Quality control involves the monitoring of data processing steps from raw data to processed/completed datasets (including the steps of sample normalization, batch correction, transformation and handling of missing values) and the simultaneous assessment of quality metrics. Concomitantly, a data quality-centered workflow would facilitate consistent processing to minimize technical variance and interference. Additionally, such a workflow would ideally identify systematic trends and deviations (systematic errors) and remove these prior to data interpretation to enable sound biological information retrieval. To offer a tool to the community that addresses this gap, MatrixQCvis was developed.

While static reports may, for example, be well-suited for documentation and more amenable to automated interpretation, an advantage of interactive interfaces is the ability to immediately explore the data using multiple plots and parameter choices and to generate and readily pursue hypotheses. Interactivity also may help users in making decisions whether to keep or drop samples while doing quality assessment. Since MatrixQCvis is based on shiny (see Fig.  1c and d), it requires little programmatic interaction to start the quality assessment and is, thus, suitable for anyone who would like to analyze data quality in a fast, efficient and standardized manner. MatrixQCvis enables users to explore data quality of rectangular datasets that are represented as a SummarizedExperiment object (see Fig.  1b) interactively and reproducibly. The interface to the shiny interface is initiated with a single call to the shinyQC function. A SummarizedExperiment object can be passed to the shinyQC call directly or loaded via an upload interface. Using domain-and study-specific knowledge, MatrixQCvis guides the user to downsample datasets on the per-sample and per-feature level.

For data entities where missing values are present in the assay structure the interface will load specific interfaces to assess the data quality with respect to missing values, e.g. tab panels to visualize the number of missing values across samples or sets of missing values across sample types, and a user interface to control imputation.

Besides the interactive quality exploration, MatrixQCvis enables users to download all plots and to generate markdown/HTML reports from within the shiny interface.

## 3 Conclusion

The shiny application MatrixQCvis generates interactive data quality workflows and facilitates to monitor data quality along the major data processing steps via several commonly applied data quality metrics and visualizations. It enables users to create a dynamic, easy-to-share and easy-to-store report using user-specified settings. MatrixQCvis can be integrated into existing workflows and provides a means to scrutinize the data quality of rectangular datasets in a fast, efficient and standardized manner.

## Supplementary Material

btab748_supplementary_data
